# Circulating Cytokines Reflect the Etiology-Specific Immune Environment in Cirrhosis and HCC

**DOI:** 10.3390/cancers14194900

**Published:** 2022-10-07

**Authors:** Boris J. B. Beudeker, Zwier M. A. Groothuismink, Annemiek A. van der Eijk, Jose D. Debes, Andre Boonstra

**Affiliations:** 1 Department of Gastroenterology and Hepatology, Erasmus MC University Medical Center, 3015 CN Rotterdam, The Netherlands; 2 Department of Viroscience, Erasmus MC University Medical Center, 3015 CN Rotterdam, The Netherlands; 3 Department of Medicine, University of Minnesota, Minneapolis, MN 55455, USA

**Keywords:** cirrhosis, liver disease etiology, liver cancer, cytokines

## Abstract

**Simple Summary:**

Chronic liver diseases commonly cause severe scarring of the liver (cirrhosis) and liver cancer. The eventual progression of this scarring process to liver cancer is influenced by a variety of factors, including inflammatory cytokines (soluble mediators of cell communication). In order to increase our understanding of these mediators we studied them in the blood of patients with hepatitis B (HBV), hepatitis C (HCV), alcoholic liver disease (ALD) and non-alcoholic fatty liver disease (NAFLD) patients with liver cirrhosis. We studied more than a 100 cytokines in up to 400 patients. We discovered that patients with cirrhosis had a vast upregulation of a wide variety of immune mediators, which stimulated inflammation and were linked with tumor-promoting roles. In contrast to prevailing assumptions, each type of underlying liver disease exhibited a unique immune mediator profile in blood, which is likely to impact how we will study these markers in the future. Patients with HBV cirrhosis had the largest number of upregulated inflammation-inducing mediators, compared to HCV, ALD and NAFLD. Next, we related blood immune mediator levels with liver cancer. To do so, we studied cytokine profiles in patients with small tumors, thus, those qualified for surgical curative strategies. We observed unique sets of cytokines in serum in each liver cancer group, indicating a role for these mediators in detecting liver cancer. In conclusion, our findings underscore the impact of different liver diseases on circulating immune mediators. Future studies that aim to study them as diagnostic tools will need to correct for this important effect accordingly.

**Abstract:**

Background and Aims: Chronic liver disease—from any etiology—can progress to fibrosis, cirrhosis and hepatocellular carcinoma (HCC). The progression of liver cirrhosis to the end stages of disease is influenced by a variety of factors, including inflammatory cytokines. We pursued a study of cytokine-mediated inflammatory responses in hepatitis B (HBV), hepatitis C (HCV), alcoholic liver disease (ALD) and non-alcoholic fatty liver disease (NAFLD) patients with liver cirrhosis. Methods: Immune profiles were determined through the serum multiplex profiling of >100 cytokines in a 188 cirrhotic patients, 35 healthy controls and 196 early-stage HCC patients. Results: Patients with liver cirrhosis exhibited a vast upregulation of proinflammatory cytokines (*p* < 0.0001), including those with pro-oncogenic features, when compared to healthy individuals. In contrast to prevailing assumptions, each etiological cause of cirrhosis exhibited a unique cytokine profile in blood. Regardless of antiviral therapy, HBV cirrhosis patients had the largest number of upregulated proinflammatory mediators, compared to HCV, ALD and NAFLD (*p* < 0.0001). To further evaluate the etiology-dependent modulation of cytokine response in relation to liver cancer, we studied cytokine profiles in early-stage HCC patients strictly stratified by underlying liver disease. We observed unique sets of differentially expressed cytokines in each cohort of early-stage HCC patients of different cirrhosis etiologies. Conclusions: Our findings, therefore, underscore the importance of stratification by the etiological cause of liver cirrhosis in immune-based studies.

## 1. Introduction

Chronic liver disease is a common cause of global mortality. The primary causes of liver injury in patients are alcoholic liver disease (ALD), non-alcoholic fatty liver disease (NAFLD) and chronic viral infections with hepatitis C (HCV) or hepatitis B (HBV). These chronic injuries lead to liver fibrosis or cirrhosis and place patients at high risk of developing hepatocellular carcinoma (HCC) [1]. The immunology behind the progression of liver cirrhosis to the later stages of liver disease is controlled by a variety of factors, including inflammatory cells and cytokines [2,3,4,5]. Cytokines encompass a pleiotropic set of proteins, including a wide variety of interleukins, interferons, chemokines and growth factors. These circulating molecules are deeply involved in the progression and pathogenesis of cirrhosis as they recruit immune cells with regenerative, inflammatory and immunosuppressive features [3,4,5]. To date, a large number of cytokines have been discovered, but their importance in liver cirrhosis is not well understood. However, this information is relevant, since it may lead to a better understanding of the role of the immune system in liver disease progression and HCC development, and the levels of cytokines may identify specific disease stages. In a recent study, we reported on a serum cytokine panel that identified HCV patients who would develop HCC within 1–2 years. This cytokine panel was able to predict the development of HCC before the tumor was visible by imaging [2]. Our study focused on individuals with HCV only; it is unclear whether these findings could be extrapolated to patients with different etiological causes of cirrhosis. Different etiologies leading to cirrhosis and HCC are likely to expose unique immunopathologies with characteristic immune responses, as is reflected by etiology-specific responses to immune therapy and distinct T-cell populations in NAFLD- and HBV-affected livers [6,7]. The interest in cytokines as novel predictive HCC biomarkers and the growing potential of immune-based therapies for cirrhosis and HCC necessitates a more detailed study on the disease parameters influencing the circulating cytokine profiles in liver disease. Therefore, we performed a comprehensive evaluation of blood cytokine patterns in cirrhotic patients with distinct etiologies, and we evaluated circulating cytokines and their correlation to early-stage HCC patients stratified by HBV-, HCV-, and ALD- and NAFLD-associated cirrhosis.

## 2. Material and Methods

### 2.1. Study Participants

A retrospective cohort was established with cirrhosis and HCC patients visiting Erasmus Medical Center between 1 January 2009 and 31 December 2020. Patients were identified using a prospectively maintained pathology database and health care insurance registries.

#### Cirrhotic Patients

All participants had cirrhosis, confirmed by biopsy (Metavir) or liver transient elastography (Fibroscan^®^). Liver cirrhosis patients secondary to chronic infections with HCV or HBV, alcohol consumption and NAFLD, with a WHO performance score of 0, Child–Pugh A or B, and no history of HCC since blood collection, served as our cirrhosis study cohort and served as controls for HCC development. Chronic HBV patients with cirrhosis were serum HBV surface antigen (HBsAg)-positive for more than 6 months, and both nucleoside analog treated and untreated patients were included. Chronic HCV patients were anti-HCV positive and HCV RNA-positive and were not on treatment with direct acting antivirals. Patients with alcoholic liver disease had persistent histopathological steatohepatitis and a daily intake of >40 g ethanol for men and >30 g for women for more than 10 years, in the absence of other triggers of liver damage. Patients with NAFLD had persistent histopathological liver steatosis and lacked any evidence of ongoing or recent consumption of significant quantities of alcohol, medication or other inducers of steatosis. Cases were excluded if they showed evidence of decompensated cirrhosis; infection with a second hepatitis virus, hepatitis D virus, hepatitis E virus or human immunodeficiency virus; presence of auto-immune liver disease, hemochromatosis, Wilson’s disease, antitrypsin alpha-1 deficiency, primary biliary cholangitis, primary sclerosing cholangitis, documented clinical history of immunomodulatory drugs, other malignancies; or with ageing-associated debilitating cardiovascular diseases, autoimmune diseases and hyperplastic syndromes. A complete medical history was obtained for each patient, including antiviral therapy, alcohol consumption, and signs of type II diabetes. Data on smoking history was not consistently documented in the electronic health records and, therefore, not registered in this study.

Healthy individuals were included at the outpatient clinic after having undergone screening for benign intrahepatic lesions and had no relevant inflammatory, fibrotic or oncologic history.

HCC patients were diagnosed according to the diagnostic criteria of the American Association for the Study of Liver Disease. Only HCCs that were confirmed by histology or diagnosed via contrast-enhanced imaging were included. Barcelona Clinic Liver Cancer (BCLC) staging system was evaluated on all patients in the database, and only patients with early-stage disease, i.e., BCLC stage 0 and stage A and with a serum sample available in our biobank were included. Patients with liver cirrhosis and HCC, secondary to chronic infections with HCV or HBV, alcohol consumption and NAFLD were compared in terms of clinical characteristics and the exploratory analytes.

### 2.2. Sample Collection and Processing

Archived biobank serum of patients was collected during visits at the outpatient and isolation was done under aseptic conditions within clean room facilities, in accordance with Good Manufacturing Practice regulations.

### 2.3. Ethics

All patient samples and data used in this study were collected in the context of routine clinical patient care and the Institutional Review Board of the Erasmus Medical Center in Rotterdam approved of the use of these data and samples (METC-2017-1140 and MEC-2020-0383). The biobanked sera, and the subsequent use of these data for the study of circulating immune profiles was permitted by the board, which also waived the need for informed consent.

### 2.4. Assay Methods

Circulating cytokines were measured using the Bio-Plex platform, specifically the Bio-Plex human cytokine 47-Plex panel; the Bio-Plex human chemokine 40-Plex panel; and Single-Plex kits for pentraxin-3, MMP-1, MMP-2 and IL-6Rα (Bio-Rad Laboratories, Hercules, CA, USA). In total, 93 experimental analytes were tested in 4 different panels. Since 19 analytes were present in two or more panels, our analysis was conducted with 74 analytes.

The multiplex protein assays were performed, as described by the manufacturer. In short, cytokine standards were determined in duplicate. Serum samples were diluted four times with sample diluent. Fifty µL anti-cytokine conjugated beads were plated in a 96-well; washed twice; and 50 µL cytokine standards, kit control or serum samples were added and incubated for 30 min on a shaker. Plates were washed three times with 100 µL Bio-Plex wash buffer, then 25 µL detection antibody was added and plates were incubated for 30 min on a shaker. Next, the wash steps were repeated and 50 µL streptavidin-phycoerythrin was added. Finally, the plates were incubated for 10 min and washed three times, and the beads were suspended in Bio-Plex assay buffer. Data were acquired on a validated and calibrated Bio-Plex 200 system (Bio-Rad) by measuring the fluorescent signal of the fluorescent reporters. The Bio-Plex Manager 6.2 software (Bio-Rad) was used for data analysis. The standard curves were calculated using five parameter logistic regression analysis with default automated weighing. The highest and lowest reliable values of the standard curve of every individual analyte was selected as lower and upper limits of quantification. Undetectable values were substituted by dividing the lower limit by two.

To assure reproducibility of the measurements between different batches, sera of healthy controls and HCV patients were pooled, aliquoted, and included at different positions on each assay plate. In addition, a technical replicate provided by the manufacturer was also included on each plate. The coefficient of variation (%CV) was calculated for each as [SD/mean] × 100, and five markers with %CV > 30 were excluded (FGF-basic, GM-CSF, IL-9, CCL7/MCP-3 and CCL19/MIP-3β). The limit of detection of the eligible analytes are presented in Supplementary Tables S1 and S2. Serum levels of AFP (ng/mL), PIVKA-II (DCP) (AU/mL) and IL-6 (pg/mL) were measured on a Lumipulse G1200 (Fujirebio Inc., Tokyo, Japan), using the LUMIPULSE G AFP-N kit (Fujirebio), LUMIPULSE G PIVKA-II kit (Fujirebio) and LUMIPULSE G IL-6 kit, respectively, according to the manufacturer’s instructions.

### 2.5. Statistical Analysis

Serum biomarker levels across cirrhotic patients, HCC patients and healthy controls were statistically tested. The Mann–Whitney Wilcoxon Test, Kruskall–Wallis test, Chi-square or the Pearson correlation were applied, when appropriate. False discovery rate was determined using the two-stage linear step-up procedure of Benjamini, Krieger and Yekutieli with Q = 1%. Multiple comparisons among the cirrhotic patients, HCC patients and healthy individuals were corrected using the Holm–Bonferroni or Bonferroni method. All statistical tests were two-sided and *p* < 0.05 was considered statistically significant. R studio (version 1.4.1717) and GraphPad Prism (version 8) were used to perform statistical analyses.

## 3. Results

### 3.1. Pro-Inflammatory and Pro-Oncogenic Cytokine Patterns in Patients with Compensated Liver Cirrhosis

**Figure 1:**
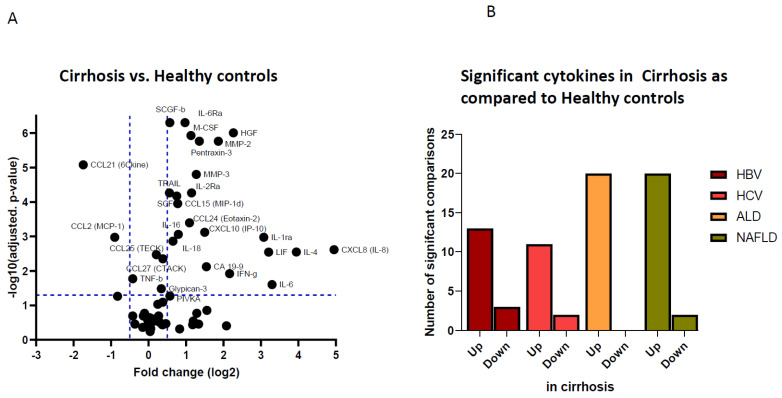
Protein expression of pro-inflammatory and immune regulating cytokines in cirrhosis vs. healthy controls. Vulcano plot of the log2 ratio vs. adjusted *p*-value of the differential levels of serum cytokines comparing healthy controls (N = 35) vs. liver cirrhosis (N = 188) (**A**). Bar plots show the number of significant Mann–Whitney U tests for the comparison between healthy controls and HBV (N = 47), HCV (N = 47), alcohol (N = 46) and NAFLD (N = 48) compensated liver cirrhosis patients (**B**). ‘Up’ means significant upregulation in cirrhosis and vice versa ‘down’ is significant downregulation in cirrhosis, as compared to healthy controls. All presented *p* values are adjusted for multiple comparisons using Holm–Bonferroni method.

In order to study the cytokine milieu in cirrhosis, we conducted multiplex protein assays on the sera of 188 patients with compensated cirrhosis secondary to HBV or HCV infections, NAFLD or ALD and compared them to sera from 35 healthy individuals (Table 1). The analytes included factors related to inflammation, cancer and immune response, providing a wide array of pathogenic mediators. Out of 69 analytes evaluated, 16 were undetectable in serum, among them β-NGF, GM-CSF, IFN-α2, IL-1α, IL-2, IL-3, IL-5, IL-7, IL-10, IL-12(p40), IL-12(p70), IL-13, IL-15, IL-17A, CCL7/MCP-3 and CCL20/MIP-3α (Supplementary Table S1). Fifty-three analytes were detectable in serum of cirrhotic patients or healthy individuals. A comparison of serum concentrations showed that 24 out of 53 circulating—largely proinflammatory—cytokines were significantly different in those with liver cirrhosis, as compared to healthy individuals. Of interest, we found differential levels in CXCL8 (IL-8), which showed up to 30-fold increased concentration in cirrhotic patients, as compared to controls, up to 10-fold increases of IL-4, IL-6, LIF, IL-1ra, HGF and CXCL10. We also observed lower serum levels of CCL21 and CCL2 (Figure 1A). As shown in Figure 1B, significant differences were observed in the levels of serum cytokines in all four etiologies, as compared to healthy controls, with the majority of them being upregulated in cirrhotic patients, compared to controls. A number of these analytes were differentially expressed related to liver disease, including proteins with inflammatory and immunomodulatory activities, such as CCL15, pentraxin-3, TRAIL and MMP-3, among others, as well as the growth factors M-CSF, SCF, SCFG-β and HGF (Supplementary Figure S1). These circulating mediators all have pleiotropic effects, but specific intrahepatic and tumor-promoting functions have been ascribed to them: circulating CCL15 has been associated with presence of suppressive monocytes in the liver; MMP-3 is linked to HGF-induced HCC invasion [5,8], while the growth factors M-CSF, SCF, SCFG-β and HGF are all known to contribute to tumor progression in a broad spectrum of tumors [9]. These findings clearly demonstrate a dramatic change in the cytokine profile in blood of liver cirrhosis patients, suggestive for activation of pro-inflammatory, immunomodulatory and HCC-promoting immune pathways.

### 3.2. Etiological Cause of Cirrhosis Associates with Unique Immune Profiles in Blood

**Figure 2:**
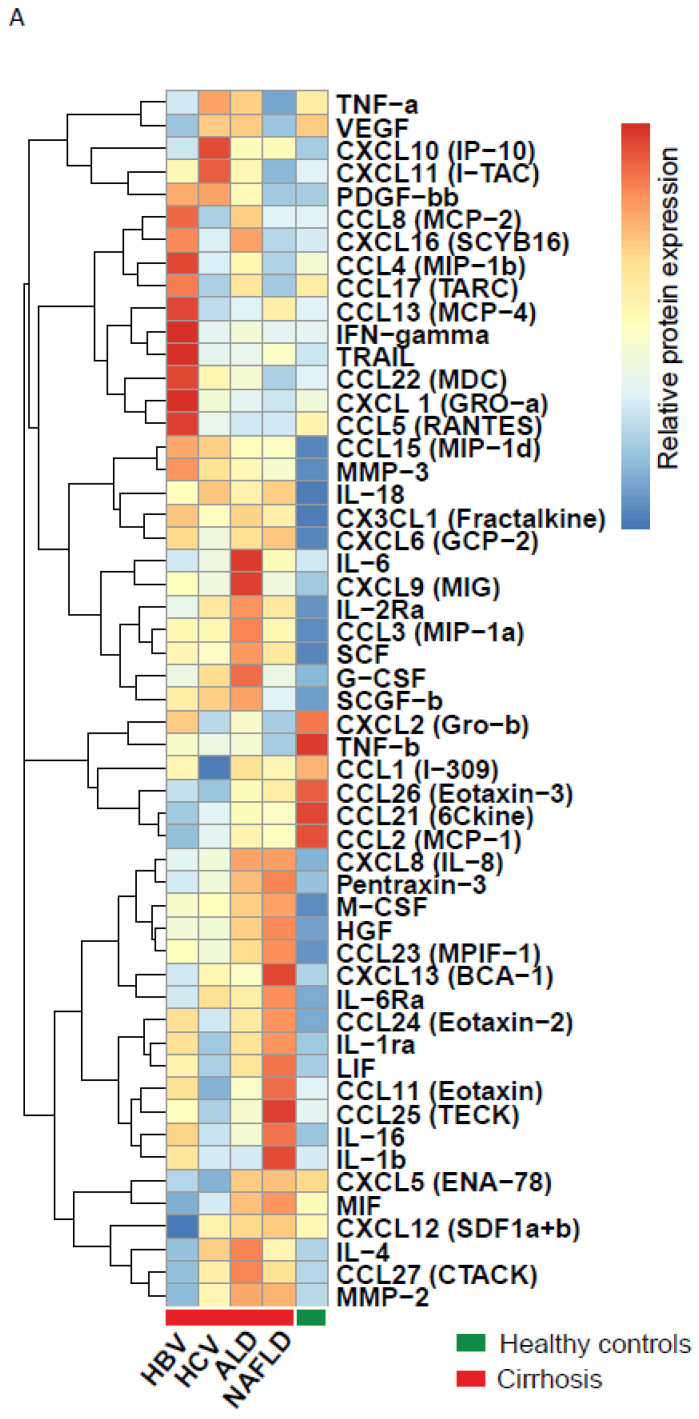

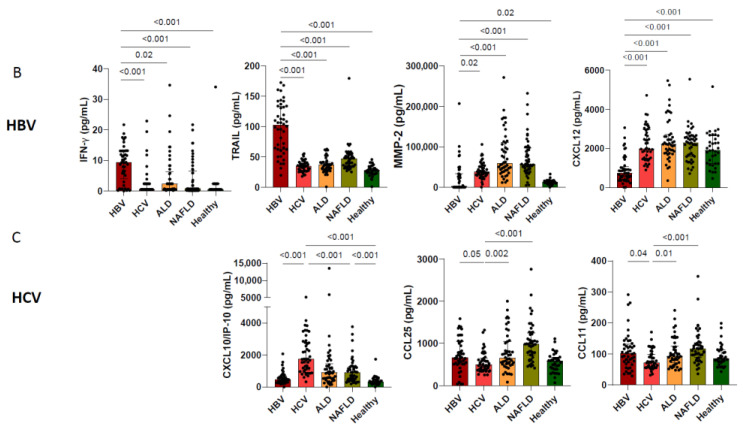

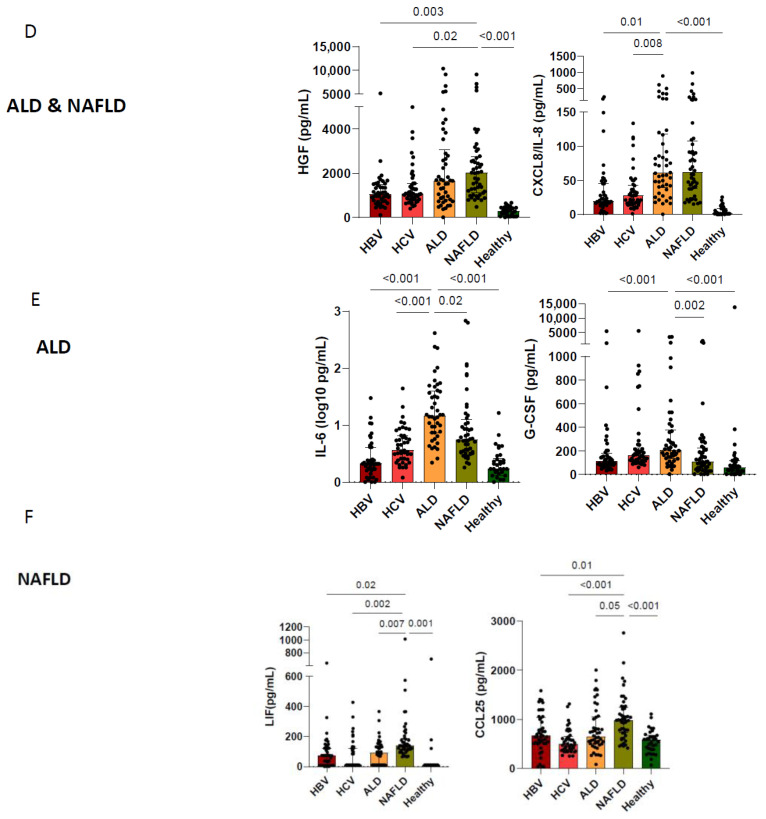
Heatmap of median protein levels with hierarchical clustering of the 53 significant circulating analytes in healthy controls and HBV, HCV, ALD and NAFLD cirrhosis patients (**A**). Scatter dot plots with a selection of significantly different circulating cytokines levels in cirrhosis per etiology: HBV (**B**) HCV (**C**), ALD (**D**,**E**) and NAFLD (**D**,**F**). All presented *p* values are adjusted for multiple comparisons using the Bonferroni method.

Since we demonstrated that the serum cytokine levels differed between samples from cirrhotic patient and healthy individuals, we decided to stratify the cirrhotic samples for different etiological causes (HBV [N = 47], HCV [N = 47], ALD [N = 46] and NAFLD [N = 48]). Heat map analysis identified highly distinctive profiles in cytokine expression between groups, with 47 significantly expressed cytokines in one of the cirrhosis etiologies, and at least four immunological profiles were evidently observed (Figure 2A and Supplementary Table S2).

Upon detailed assessment, cytokine levels and cirrhosis etiology revealed that HBV-cirrhosis—regardless of antiviral therapy—had a 3-fold increase in IFN-γ, TRAIL and CXCL1 (GRO-α) levels and up to 3-fold decrease in MMP-2, CXCL12, IL2RA, IL-6Rα, CCL2 (MCP1) and CCL21 (6kine) levels (Figure 2B). HCV-cirrhosis was characterized by a 3-fold increase in CXCL10 but not IFN-γ. The serum levels of CCL25 and CCL11 were decreased in HCV cirrhosis, in comparison to the other cirrhotic groups but not to healthy controls (Figure 2C). The serum profiles of cytokines of ALD- and NAFLD-cirrhosis both displayed relatively high HGF and CXCL8 serum levels when compared to the HBV- or HCV-cirrhosis group (Figure 2D). Moreover, the ALD group was characterized by higher serum levels of IL-6 and G-CSF (Figure 2E), while the NAFLD group showed higher levels of LIF and CCL25 (Figure 2F). These findings demonstrate that although the serum profiles of distinct cirrhotic patients display some common characteristics, etiology-specific patterns are observed that are highly distinct and reflective of the ongoing immune response. These results compel us to rethink the importance of etiology when studying cirrhosis and HCC and may explain the poor reproducibility of biomarker studies in liver disease.

### 3.3. Cirrhosis Etiology Impacts Peripheral Immune Patterns of Early-Stage HCC

**Figure 3:**
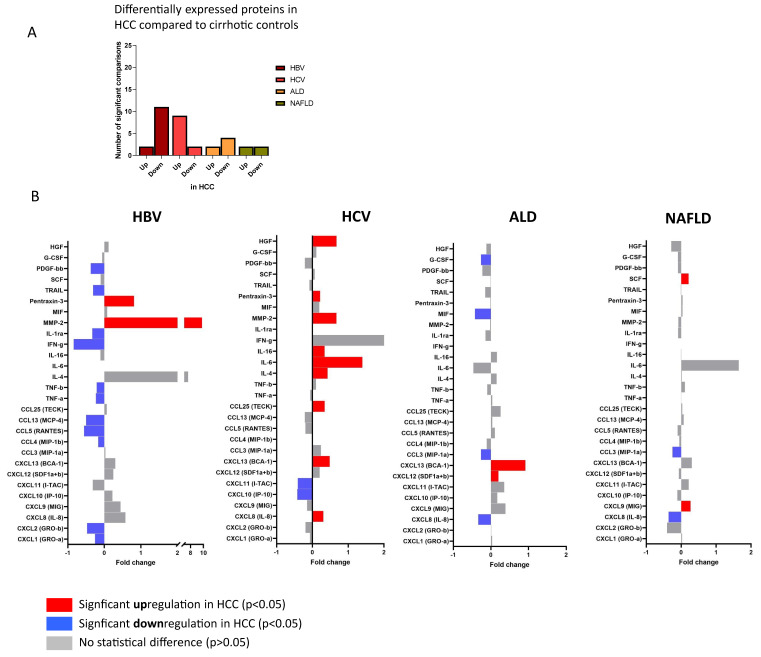

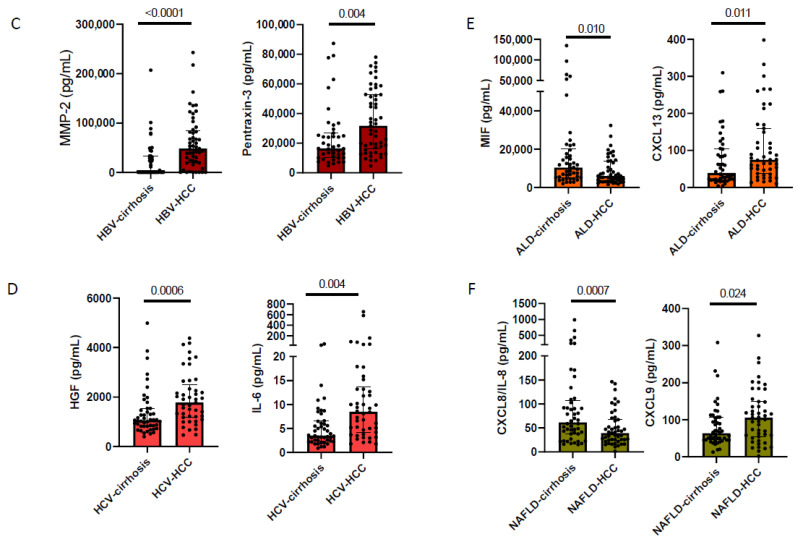
Bar plots show the number of significant Mann–Whitney U test for the comparison between HBV-HCC and HBV-cirrhosis, HCV-HCC and HCV-cirrhosis, ALD-HCC and ALD-cirrhosis, NAFLD-HCC and NAFLD-cirrhosis (**A**). Fold change plot of cytokine, chemokine and growth factor levels in HCC subjects, normalized for the median levels in cirrhosis subjects. Bar graphs represent the normalized median HCC analyte levels relative to the normalized median cirrhosis analyte levels. Red bars: analyte levels significantly greater in patients with HCC than in patients with cirrhosis (upregulation). Blue bars: analyte levels significantly lower in HCC than cirrhosis patients (downregulation) (**B**). Scatter dot plots of the top-ranked circulating cytokines, chemokines and growth factor levels in HCC and cirrhosis per etiology: HBV (**C**) HCV (**D**), ALD (**E**) and NAFLD (**F**).

Ourselves and others have previously reported on specific circulating cytokine profiles that may be indicative of the presence of HCC at a very early stage of its development, suggesting a potential role for blood immune mediators as biomarkers [2,10,11]. However, given our findings that liver cirrhosis and underlying disease etiology have a major impact on the circulating immune profile, we thought it important to assess whether the etiological cause of cirrhosis affected cytokine-based analytes of early-stage HCC. Therefore, we compared cirrhosis cases without HCC (N = 188) with those with HCC (N = 196) strictly classified by underlying liver disease (Table 1). Across the four etiologies (NAFLD, ALD, HBV, HCV), we found that 28 out of 53 cytokines showed distinct levels in serum of patients with HCC, compared to cirrhotic patients without HCC irrespective of etiology of liver disease (Figure 3A,B). Interestingly, the number of differentially modulated cytokines showed an inverse pattern between viral-related and non-viral-related liver disease in individuals with HCC. Indeed, in sera from cirrhosis patients without HCC, the ALD and NAFLD groups showed a higher degree of differentially expressed cytokines, compared to the viral hepatitis groups (Figure 1B); in contrast, in sera from cirrhotic patients with HCC, the HBV and HCV groups showed a higher number of differentially expressed cytokines, compared to the groups with metabolic causes (Figure 3A), suggesting an alternative path to oncogenesis between infectious and non-infectious etiologies. Direct comparisons between cirrhosis and HCC groups revealed that there was no common overall profile associated with HCC, but rather etiology-specific differences (Figure 3B). Sera from HBV-HCC patients were characterized by higher MMP-2 and pentraxin-3 levels, with a trend toward lower cytokine and chemokine concentrations, when compared to HBV patients with cirrhosis (Figure 3B,C). Serum from HCV-HCC patients showed higher levels of MMP-2 and pentraxin-3 as well, but, in addition, exhibited increased levels of IL-6 and HGF in HCC compared to HCV-cirrhosis patients (Figure 3B,D). NAFLD- and ALD-HCC showed lower numbers of cytokines that differed from their respective controls, as compared to the HBV and HCV groups. Both NAFLD-HCC and ALD-HCC showed lower levels of CXCL8 (IL-8) and CCL3, compared to their respective cirrhotic counterparts (Figure 3B). The most differentially expressed ALD-HCC markers were upregulated CXCL13 and downregulated MIF; while NALFD-HCC was characterized by lower CXCL8 and higher circulating CXCL9 levels (Figure 3E,F). Overall, these findings expose clear differences in immune analytes in HCC, based on the etiological difference of liver disease, highlighting the importance of stratifying for etiology when assessing immune-based analytes for consideration as potential biomarkers.

## 4. Discussion

Liver cirrhosis is an inflammation-driven disease and an important cause of global morbidity and mortality. Here, we studied in detail the cytokine-based immune response in patients with HBV-, HCV-, ALD-, and NAFLD-associated liver cirrhosis, as well as the differential variation of these circulating cytokine signatures in patients with early-stage HCC. We found that liver cirrhosis had a profound impact on the circulating immune profile and was associated with a wide repertoire of pro-inflammatory and HCC-promoting cytokines. We observed etiology-specific cytokine responses with up to 47 significantly augmented cytokines in HBV, HCV, ALD or NAFLD. In order to study the impact of these findings, we also studied the cytokine-based immune response in liver cirrhosis patients with early-stage HCC patients. Following strict stratification, we confirmed the important immunologic role of the etiological cause of cirrhosis, as we observed etiology-specific immune profiles that strongly correlated with the presence of early-stage HCC.

In recent years, elegant immunological studies found that cirrhosis etiology can influence local tumor immune responses [6,12]. These studies inspired us to study the complexity of the cytokine profiles in cirrhotic patients. When evaluating cirrhotic patients, the most obvious change was seen for CXCL8, which showed up to 30-fold increased levels in serum of cirrhotic patients, as compared to healthy individuals. CXCL8 is a multipotent molecule with angiogenic and inflammatory properties, it is produced by a wide variety of cells, such as monocytes, macrophages and fibroblast, and it is associated with leaky gut-induced inflammation, all of which suggest that CXCL8 could be a potential factor for further investigation in its role of cirrhosis development [13,14,15]. The high levels of CXCL8 and MIF are also suggestive of involvement of tumor-associated macrophages. These cells are a well-known component of the tumor microenvironment and abundantly infiltrate HCC microenvironment. A co-culture study linked macrophage-activating CXCL8 with HCC growth in vitro [16]. However, in vivo studies on the mechanisms of these markers are lacking We also observed increased expression across all etiologies of cirrhosis in growth factors related to HCC, as well as evasion-associated growth factors, such as HGF, SCGF-BB and SCF, and inflammation-driven mediators, such as pentraxin-3, CCL15, MMP-3 and TRAIL [5,8,17,18,19,20]. Our data are illustrative of the effect of cirrhosis on the immune response and underlines the importance of correcting for the presence of cirrhosis in immune-related studies. Within our cohort of cirrhotic patients, we found highly distinctive cytokine profiles between HBV-, HCV-, and ALD- and NAFLD-associated cirrhosis. The impact of cirrhosis etiology of serum AFP levels is well established and generally higher levels are seen in patients with HCV (Table 1) [21]. This effect was not seen for PIVKA-II (DCP) (Supplementary Figure S2). A closer look at the cirrhosis etiologies revealed that HBV-cirrhosis had the highest number of uniquely augmented cytokines with a prominent role for high levels of the NK and NKT cell cytokine IFN-γ, the apoptosis marker TRAIL and the neutrophil attracting CXCL1 [22]. HCV-cirrhosis was strongly associated with interferon-associated CXCL10 but not IFN-γ. A previous study on cytokines in cirrhosis demonstrated that HBV- and HCV-cirrhosis affected CXCL10, CXCL8, M-CSF and TNF serum levels [10]. In accordance with that publication, we found elevated levels of CXCL10, CXCL8 and M-CSF in serum of cirrhotic patients with HBV and HCV, and for the first time describe similar alterations in ALD and NAFLD cirrhosis. ALD-cirrhosis was associated with IL-6, CCL27 and G-CSF and NAFLD-cirrhosis with LIF and CCL25. In addition to these differences, the immune profiles of ALD- and NAFLD-cirrhosis were comparable with higher levels of CXCL8 and HGF, compared to patients with viral hepatitis. ALD and NAFLD are two diseases characterized by the presence of steatohepatitis, thus sharing a pathophysiological route towards inflammation, fibrosis and HCC. Despite there being different cytokines in each etiological cause of cirrhosis, cytokines are pleiotropic and functions can overlap. In our assessment, CXCL1 levels corresponded with HBV-cirrhosis, while CXCL8 was characteristic of non-viral etiologies. CXCL1 and CXCL8, as well as their receptor CXCR2, belong to the CXCR family and are suggested to activate similar pathways that mediate proliferation and angiogenesis [23]. Heatmap analysis of the significant cytokines in HBV, HCV, ALD and NAFLD show that without clustering, similar immune patterns are observed, as depicted in Figure 2. However, it is important to mention here that the data with two-way clustering is biased towards clustering of those markers with the highest expression and does not provide insight in statistical differences (Supplementary Figure S3). In short, the etiological cause of cirrhosis associates with unique immune profiles in blood and our data provide a rationale for further study of the impact of etiological causes on the immune system in cirrhosis. Indeed, understanding the involvement of these cytokines in the pathogenesis of cirrhosis may prove crucial for our understanding of the pathology of cirrhosis.

More than 90% of HCC cases will develop in a background of liver cirrhosis [24]. Therefore, the immune-pathogenesis of cirrhosis is deeply intertwined with that of HCC. There is an urgent need for novel easy-to-measure biomarkers, such as peripheral immune markers, that identify the early stages of HCC in the background of liver cirrhosis to improve disease outcomes and cure rates. Because of their broad applicability and ability to be assessed with standard expandable technology, multiple studies have addressed the role of cytokines in prediction and detection of HCC. These studies have included a variety of interleukins, chemokines, tissue modulating factors and growth factors, with no common panel identified in these studies [10,11,25,26]. We believe an important factor affecting limited reproducibility of the identified markers between these studies is likely the heterogeneity of the populations. Indeed, most studies include the mixed population of patients, such as those with and without liver cirrhosis, different BCLC scores, and cancer stages and, more importantly, different underlying causes of HCC, all of which limits reproducibility and could impact clinical applicability. The clinically most established serological biomarker, AFP, shows only a limited diagnostic performance for detecting early-stage HCC; however, when combined with age, gender, AFP-L3, and DCP (GALAD model), it shows promising diagnostic utility, in various etiologies of HCC, for early-stage HCC. Data on the utility of GALAD or other protein markers in surveillance is lagging behind; this is true for the candidate protein markers, such as glypican-3, but also immune markers, miRNA and epigenetic markers, and it is likely that in the years to come further optimization of the biomarker panels for early HCC will be reported. It would be interesting to examine whether serum cytokines may have additive predictive value in large prospective cohorts of early HCC in future studies [27].

Through a combination of highly sensitive and quantitative multiplex-based assays, we expose that cytokines are differentially up- or down-regulated in patients with early-stage HCC, as compared to cirrhotic patients, depending on their cause of liver disease being HBV, HCV, ALD or NALFD. The function of these markers in early-stage HCC is currently unclear and our study is not powered or designed to assess them as biomarkers. However, we provide important clues to the potential differential expression of these analytes related to underlying liver disease. Taken together, by studying circulating cytokines strictly stratified by underlying liver disease, our study provides important clues into the immunology of liver cirrhosis and the etiological cause of cirrhosis etiology. This is important in the context of recent advances in immune-based therapies and predictive biomarkers in patients with cirrhosis and HCC, and our data now provide a rationale for stratification of patients with HCC and cirrhosis according to the underlying etiology of liver disease.

## 5. Conclusions

Liver cirrhosis has a profound impact on the levels of circulating immune mediators and associates with a wide repertoire of pro-inflammatory and HCC-promoting cytokines. Among the patients with liver cirrhosis, we observed significantly enhanced circulating cytokine levels in HBV, HCV, ALD and NAFLD, which underlines the impact of cirrhosis etiology on the circulating immune profile. We also studied the cytokine-based immune response in liver cirrhosis patients with early-stage HCC patients. Following strict stratification, we confirmed the important immunologic consequences of the etiological cause of cirrhosis, as etiology-specific immune profiles were observed that strongly correlated with the presence of early-stage HCC. Future prospective studies will have to unravel whether monitoring the circulating immune response to predict the development of HCC and cirrhosis etiology deserve more attention in studies that aim to unravel the mysteries of the immune system in fibrosis and HCC.

## Figures and Tables

**Table 1:** Characteristics of the HBV-, HCV-, Alcohol- or NAFLD-associated early-stage hepatocellular carcinoma (HCC) and liver cirrhosis patients (WHO PS 0, Child-Pugh A-B). Barcelona Clinic Liver Cancer prognosis and treatment stage; BCLC.

		HBV-Associated		HCV-Associated		Alcohol-Associated		NAFLD-Associated		Healthy
		HCC	Cirrhosis	HCC	Cirrhosis	HCC	Cirrhosis	HCC	Cirrhosis	Control
**N=**		54	47	47	47	47	46	48	48	35
**Age (years)**	Median (IQR)	62 (15)	54 (15)	60 (11)	53 (12)	68 (11)	58 (12)	72 (12)	56 (18)	58 (5)
**Gender**										
**Female**		12 (22%)	9 (20%)	6 (13%)	13 (28%)	12 (16%)	21 (46%)	18 (37%)	24 (50%)	26 (74%)
**Male**		42 (78%)	37 (80%)	40 (87%)	34 (72%)	35 (74%)	25 (54%)	30 (63%)	24 (50%)	9 (26%)
**Ethnicity**										
**African**		7 (13.0%)	8 (17.4%)	6 (13.4 %)	2 (4.3%)	0 (0.0%)	1 (2.2%)	2 (4.3%)	5 (10.4%)	0 (0%)
**Asian**		13 (24.1%)	11 (23.9%)	3 (6.5%)	0 (0.0%)	1 (2.1%)	0 (0.0%)	1 (2.1%)	0 (0.0%)	0 (0%)
**Caucasian**		29 (53.7%)	22 (47.8%)	32 (69.6%)	35 (74.5%)	45 (95.7%)	41 (89.1%)	41 (87.2%)	41 (85.4%)	35 (100%)
**Other**		5 (9.3%)	5 (10.9%)	5 (10.9%)	10 (21.3%)	1 (2.1%)	4 (8.7%)	3 (6.4%)	2 (4.2%)	0 (0%)
**Tumor stage**										
**BCLC 0**		11 (20.3%)	0 (0.0%)	11 (23.9%)	0 (0.0%)	3 (6.4%)	0 (0.0%)	5 (10.6%)	0 (0.0%)	na
**BCLC A**		43 (79.7%)	0 (0.0%)	35 (76.1%)	0 (0.0%)	44 (93.6%)	0 (0.0%)	42 (89.4%)	0 (0.0%)	na
**Antiviral therapy**		37 (67%)	36 (78%)	0 (0.0%)	0 (0.0%)	na	na	na	na	na
**Liver cirrhosis**		54 (100%)	46 (100%)	46 (100%)	47 (100%)	47 (100%)	46 (100%)	48 (100%)	48 (100%)	0 (0%)
**AFP (ng/mL)**	Median (IQR)	9 (47)	4 (2)	22 (86)	10 (10)	8 (17)	5 (3)	7 (11)	4 (4)	4 (3)

## Data Availability

The data supporting our findings are available from the authors upon reasonable request.

## Supplementary Materials

The following supporting information can be downloaded at: https://www.mdpi.com/article/10.3390/cancers14194900/s1, Figure S1: Cirrhosis markers, associated with inflammation and tumor growth; Figure S2: Scatter dot plots wth serum PIVKA-II (DCP) levels in HBV-cirrhosis, HCV-cirrhosis, ALD-cirrhosis and NAFLD-cirrhosis; Figure S3: Heatmaps depicting z-scores of selected serum cytokines across patients with HBV-, HCV-, ALD- and NAFLD-cirrhosis; Table S1: Overview of undetectable cytokines with observed lower limit of detection; Table S2: Median serum levels of 53 cytokines, with corresponding lower limit of detection (LLOD), in patients with hepatitis B virus (HBV)-associated cirrhosis, hepatitis C virus (HCV)-associated cirrhosis, alcoholic liver disease (ALD)-associated cirrhosis, non-alcoholic fatty liver disease (NAFLD)-associated cirrhosis and healthy controls.

## Abbreviations

Limit of detection (LLOD), hepatitis B virus (HBV), hepatitis C virus (HCV), alcoholic liver disease (ALD), non-alcoholic fatty liver disease (NAFLD), hepatocellular carcinoma (HCC), interleukin (IL), chemokine (C-X-C motif) ligand (CXCL), chemokine (C-C motif) ligand (CCL), matrixmetalloprotease (MMP), granulocyte colony stimulating factor (G-CSF), hepatocyte growth factor (HGF), platelet-derived growth factor (PDGF), stem cell factor (SCF), stem cell growth factor (SCGF), tumor necrosis factor (TNF), interferon (IFN), macrophage migration inhibitory factor (MIF), leukemia inhibitory factor (LIF), vascular endothelial growth factor (VEGF), alpha-fetoprotein (AFP).
