# Supplementary material for: Circulating Cytokines Reflect the Etiology-Specific Immune Environment in Cirrhosis and HCC
Source: Cancers (Basel). 2022 Oct 7;14(19):4900. doi: 10.3390/cancers14194900 (PMC9563264; doi:10.3390/cancers14194900)
Supplement: Supplementary file 1 [file cancers-14-04900-s001.zip › Supplementary Figure S1.pdf]

Cirrhosis markers

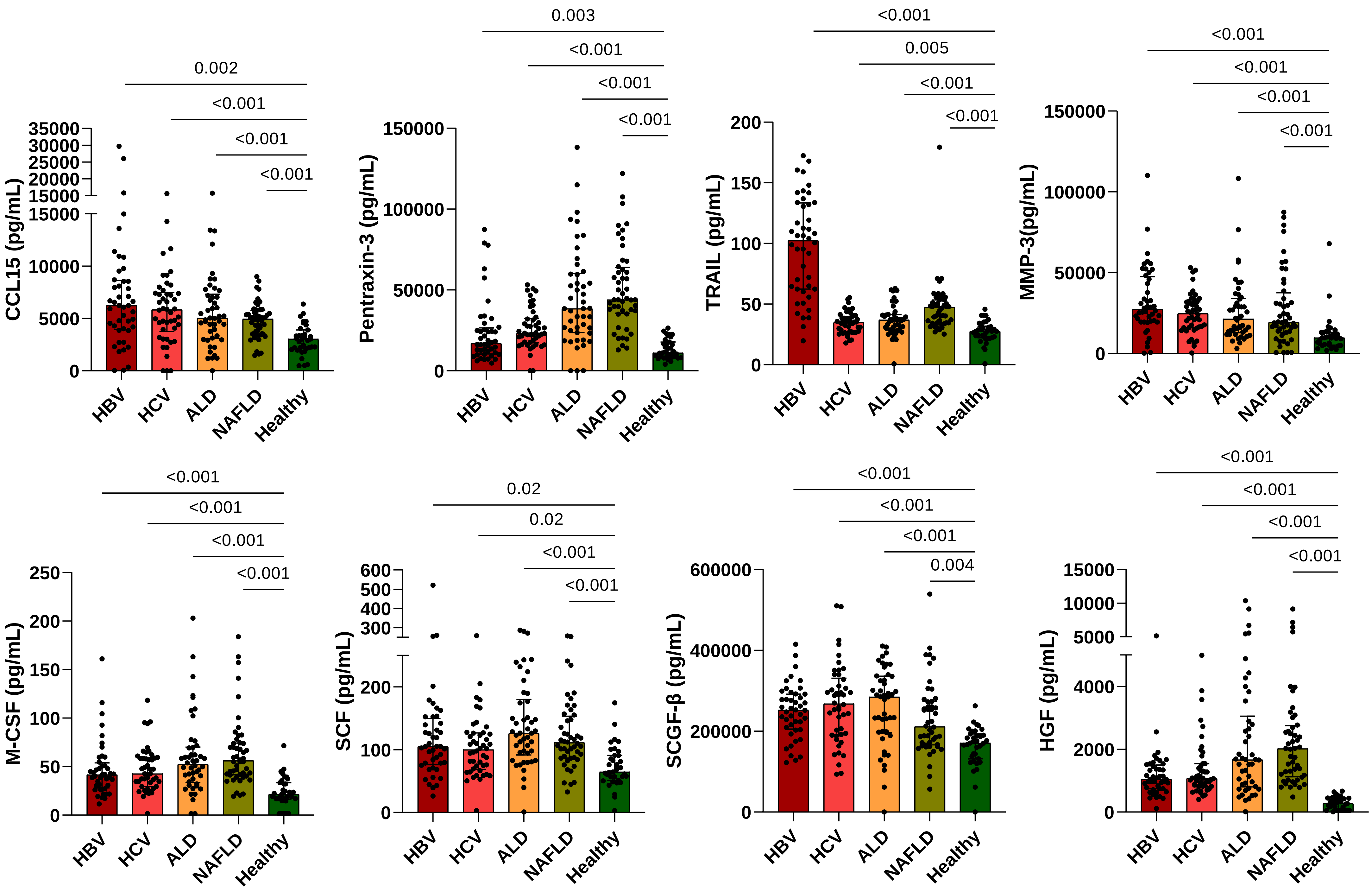

Supplementary Figure S1. Cirrhosis markers, associated with inflammation and tumor growth
