# Supplementary material for: Circulating Cytokines Reflect the Etiology-Specific Immune Environment in Cirrhosis and HCC
Source: Cancers (Basel). 2022 Oct 7;14(19):4900. doi: 10.3390/cancers14194900 (PMC9563264; doi:10.3390/cancers14194900)
Supplement: Supplementary file 1 [file cancers-14-04900-s001.zip › Supplementary Table S1, updated version.pdf]

Type of marker	Type of panel	Status	Lower limit of quotation (pg/mL)
**b-NGF**	Cytokine panel	Undetectable	0.9
**GM-CSF**	Chemokine panel	Undetectable	2.15
**IFN-a2**	Cytokine panel	Undetectable	7.715
**IL-10**	Chemokine panel	Undetectable	2.48
**IL-12(p40)**	Cytokine panel	Undetectable	44.27
**IL-12(p70)**	Cytokine panel	Undetectable	2.46
**IL-13**	Cytokine panel	Undetectable	0.665
**IL-15**	Cytokine panel	Undetectable	36.9
**IL-17A**	Cytokine panel	Undetectable	2.58
**IL-1a**	Cytokine panel	Undetectable	4.265
**IL-2**	Cytokine panel	Undetectable	1.45
**IL-3**	Cytokine panel	Undetectable	0.23
**IL-5**	Cytokine panel	Undetectable	9.495
**IL-7**	Cytokine panel	Undetectable	13.745
**CCL7 (MCP-3)**	Cytokine panel	Undetectable	5.64
**MIP-3a/CCL20**	Chemokine panel	Undetectable	0.35

Supplementary Table S1. Overview of undetectable cytokines with observed lower limit of detection.
