# Supplementary material for: Circulating Cytokines Reflect the Etiology-Specific Immune Environment in Cirrhosis and HCC
Source: Cancers (Basel). 2022 Oct 7;14(19):4900. doi: 10.3390/cancers14194900 (PMC9563264; doi:10.3390/cancers14194900)
Supplement: Supplementary file 1 [file cancers-14-04900-s001.zip › Supplementary Table S2.pdf]

Cytokine		HBV-associated cirrhosis		HCV-associated cirrhosis		ALD-associated cirrhosis		NAFLD-associated cirrhosis		Healthy control		
	LLOD	Median	IQR	Median	IQR	Median	IQR	Median	IQR	Median	IQR	P-value
**CCL1 (I-309)**	6.95	53.86	36.0825-98.52	43.86	29.16-65.54	55.635	39.29-76.9925	53.695	35.01-67.985	57.78	44.53-84.77	0.050216
**CCL11 (Eotaxin)**	7.87	102.61	72.935-131.1925	72.54	55.71-99.31	93.295	71.515-126.0825	117.21	89.605-135.9525	85.04	75.59-113.93	0.000133
**CCL13 (MCP-4)**	0.55	77.225	34.4525-164.92	34.11	15.98-55.83	38.8	21.04-61.7725	53.59	18.8675-89.2375	38.57	24.67-62.21	0.000161
**CCL15 (MIP-1d)**	2.4	6246.425	3979.2375-8910.4825	5871.9	3953.68-7699.11	5037.08	3096.2325-7318.075	4947.705	3531.4375-5773.39	3037.58	2124.89-3900.84	<0.0001
**CCL17 (TARC)**	4.09	178.07	79.8625-343.38	74.3	51.57-141.02	138.675	57.9025-233.985	69.84	20.12-191.605	134.06	64.24-195.39	0.017858
**CCL2 (MCP-1)**	0.44	45.89	28.9875-58.74	64.02	40.39-95.15	84.13	54.51-126.9875	78.025	52.0525-106.4375	122.46	95.64-145.53	<0.0001
**CCL21 (6Ckine)**	169.83	5863.155	4508.7725-12126.3225	14505.86	6522.24-30735.68	24665.09	8988.4825-53738.1275	21921.35	14059.445-33214.4775	51140.62	19530.69-71237.58	<0.0001
**CCL22 (MDC)**	2.385	851.05	437.0875-1108.1225	636.09	455.82-796.81	585.345	300.8275-882.625	469.575	355.1975-707.675	526.25	331.85-700.18	0.013222
**CCL23 (MPIF-1)**	3.79	540.065	144.13-782.8	514.54	288.07-778.71	589.935	379.3375-850.0325	637.085	330.56-925.265	410.85	294.59-611.92	0.420535
**CCL24 (Eotaxin-2)**	5.705	616.39	322.28-1041.7225	388.18	247.43-757.38	579.695	284.575-1129.175	714.12	388.915-1164.89	273.41	149-463.06	<0.0001
**CCL25 (TECK)**	40.11	681.345	510.61-1059.92	504.16	365.51-676.97	650.725	507.1075-1049.82	984.84	726.35-1248.9225	586.45	389.24-676.97	<0.0001
**CCL26 (Eotaxin-3)**	3.32	16.735	5.925-30.6575	15.58	1.635-24.76	19.96	1.65375-27.915	21.035	13.82-30.01	24.76	16.22-31.94	0.024141
**CCL27 (CTACK)**	2.085	1032.49	722.01-1446.31	1474.24	1062.65-1806.41	1767.34	1359.4325-2647.42	1547.87	1043.4175-2407.48	1110.62	840.36-1305.08	<0.0001
**CCL3 (MIP-1a)**	0.155	4.94	3.765-7.03	4.8	3.17-6.46	6.86	4.31-11.6325	5.13	3.3925-7.85	2.28	1.64-4.09	<0.0001
**CCL4 (MIP-1b)**	3.69	231.78	182.105-256.74	195.67	154.4-218.14	208.2	154.995-250.625	191.04	157.425-213.5325	202.59	181.78-217.02	0.006367
**CCL5 (RANTES)**	1.67	11795.67	4413.955-18177.26	8010.35	5063.66-11200.32	7576.145	3317.025-14295.5	7581.225	3224.5975-12481.24	9171.56	6624.11-10282.52	0.221193
**CCL8 (MCP-2)**	0.25	89.815	52.5-126.965	63.47	43.34-94.09	81.805	41.305-114.015	67.635	43.8175-101.625	67.91	46.64-118.84	0.159125
**CX3CL1 (Fractalkine)**	3.88	365.315	241.7-478.8725	294.8	211.57-397.8	356.835	284.9975-620.8175	320.955	208.205-438	140.26	114.04-274.15	<0.0001
**CXCL 1 (GRO-a)**	19.25	1196.89	809.63-1331.92	787.9	586.38-920.25	724.715	515.075-915.9475	678.22	535.015-857.715	775.59	596.79-906.09	<0.0001
**CXCL10 (IP-10)**	11.13	467.03	291.45-680.01	1730.81	1069.87-2779.23	895.35	505.26-1452.675	916.91	467.865-1262.7325	313.46	233.66-488.73	<0.0001
**CXCL11 (I-TAC)**	0.38	10.76	4.4425-20.3725	14.54	7.09-35.98	10.67	5.135-22.9525	7.295	2.67-17.8825	9	5.44-12.93	0.024214
**CXCL12 (SDF1a+b)**	19.935	743.9	452.0325-1054.4325	1976.75	1456.4-2954.02	2224.83	1772.585-2707.6325	2293.92	1605.08-2649.6175	1913.29	1228.84-2658.01	<0.0001
**CXCL13 (BCA-1)**	1.56	27.935	14.7175-48.955	43.02	24.7-66.3	39.15	20.8375-104.06	71.02	33.395-99.525	23	13.62-55.16	<0.0001
**CXCL16 (SCYB16)**	1.5	729.445	554.825-968.79	583.51	508.03-799.85	715.325	563.21-823.7575	561.895	407.6325-713.505	580.7	524.35-702.8	0.003716
**CXCL2 (Gro-b)**	21.3	345.91	193.8075-544.62	188.53	149.22-328.23	262.035	111.165-489.955	172.8	78.27-298.3375	403.59	235.18-633.17	<0.0001
**CXCL5 (ENA-78)**	232.09	1360.12	1000.0775-1908.1675	1298.73	432-2189.62	1638.82	864.27-2557.71	1647.65	1058.81-2299.1225	1618.55	567.69-2424.87	0.088037
**CXCL6 (GCP-2)**	5.635	81.61	50.9775-109.85	60.51	24.16-86.12	80.035	42.93-124.455	85.105	63.39-160.9975	31.86	5.77-76.53	<0.0001
**CXCL8 (IL-8)**	1.4	20.18	15.12-45.35	27.49	16.37-42.37	60.995	39.4675-117.525	61.63	37.57-107.67	1.295	1.295-8.74	<0.0001
**CXCL9 (MIG)**	3.45	67.865	48.795-105.955	64.1	45.5-143.02	88.245	65.1875-148.535	63.675	45.63-106.5125	54.77	42.8-80.87	0.019118
**G-CSF**	3.6	108.06	79.5-174.135	161.07	107.3-216.72	206.695	136.22-378.355	108.44	53.17-223.1925	58.26	29.28-118.24	<0.0001
**HGF**	47.06	1042.43	742.47-1480.045	1062.85	819.67-1543.87	1666.12	799.2525-3051.9175	2016.64	1124.7125-2751.2075	271.07	47.06-442.84	<0.0001
**IFN-γ**	0.35	9.37	4.565-13.1	0.53	0.53-2.375	2.375	0.53-6.23	0.74	0.175-6.65	0.53	0.485-2.375	<0.0001
**IL-16**	6.69	275.545	199.98-354.0225	154.42	102.09-212.7	203.05	149.4125-259.5725	335.09	197.26-473.76	126.45	101.12-238.75	<0.0001
**IL-18**	1.76	77.71	51.935-106.6	89.06	64.02-146.99	80.65	59.88-115.1425	87.785	64.665-149.09	53.33	35.15-63.1	<0.0001
**IL-1b**	0.54	2.215	0.54-3.98	0.54	0.49-4.52	1.395	0.49-3.705	2.96	2.18-4.63	0.54	0.49-3.58	0.002234
**IL-1ra**	72.65	635.975	469.1875-899.8625	72.455	72.455-673.2	621.6	72.455-1071.155	854.12	553.87-1140.49	72.455	66.53-72.65	<0.0001
**IL-2Ra**	4.78	107	78.23-153.345	142.47	110.61-199.67	172.28	113.2725-257.0975	143.115	101.55-208.665	62.21	35.94-82.7	<0.0001
**IL-4**	6.38	0.4	0.4-6.28	9.85	7.62-13.1	12.76	8.73-18.8125	7.315	5.41-9.12	0.52	0.48-7.83	<0.0001
**IL-6**	1.78	2.1	1.6-4.1	3.55	2.35-6.525	14.65	7.4-40.2	5.55	3.4-12.725	1.7	1.3-2.6	<0.0001
**IL-6Ra**	36.33	39898.44	24894.47-57772.115	61226.41	49158.4-73956.32	56471.865	42176.555-71559.1625	71324.735	56378.24-88766.0925	29211.21	23844.06-36910.82	<0.0001
**LIF**	10.58	74.74	7.5-126.7075	10.58	10.255-120.77	89.635	10.49875-130.56	139	116.41-186.765	10.255	10.255-10.58	<0.0001
**M-CSF**	1.595	41.39	27.01-52.825	42.61	29.56-58.94	52.385	33.7325-70.2025	56.13	41.555-74.23	21.6	16.13-33.45	<0.0001
**MIF**	74.5	3696.175	2038.605-7954.9325	5697.29	3470.67-11336.72	10398.88	5223.6975-20191.7875	11501.095	7097.165-23331.5875	8016.67	5330.09-11815.36	<0.0001
**MMP-2**	396.915	4480.37	400-38350.8	38931.58	30407.85-53589.08	59412.725	41176.6525-110854.5425	57560.7	43470.115-96849.9775	12517.56	7675.91-15695.29	<0.0001
**MMP-3**	506.75	28116.08	19741.89-47049.275	24036.32	15910.67-31849.81	21252.61	12812.1725-33947.7725	19293.665	13058.565-37536.6525	9643.19	5186.17-13101.55	<0.0001
**PDGF-bb**	21.955	1936.81	1405.32-2845.105	1967.81	1270.34-3168.39	1659.85	968.16-2479.0675	1302.65	952.9975-2111.77	1313.78	1022.71-1958.03	0.008319
**Pentraxin-3**	61.785	17541.88	10702.745-26164.335	23340.57	17739.01-32094.79	38420.495	23633.1375-60186.8625	43932.96	35651.935-63985.915	11086.81	8550.62-17811.11	<0.0001
**SCF**	3.185	104.98	73.145-147.24	100.12	69.34-126.42	126.42	91.4675-180.125	111.065	86.705-153.46	64.51	51.07-90.93	<0.0001
**SCGF-b**	250.12	251862.97	206562.61-291861.685	269894.48	191924.98-340055.33	284888.955	197492.535-336058.2325	211424.57	166061.715-273526.39	170910	131994.4-193133.07	<0.0001
**TNF-a**	3.235	34.52	26.55-48.31	39.76	26.77-53.02	38.7	32.26-55.1	32.87	20.1175-42.6425	37.92	27.51-46.42	0.185815
**TNF-b**	9.315	468.21	400.315-534.695	450.1	267.05-602.57	462.56	247.785-714.6425	402.82	306.51-537.14	601.13	354.77-682.07	0.045999
**TRAIL**	0.985	100.97	62.37-132.93	35.04	26.91-39.41	36.85	28.67-41.48	47.355	34.61-53.4975	27.6	22.3-31.75	<0.0001
**VEGF**	38.635	36.35	15-45	38.34	38.34-38.635	38.34	36.35-38.635	36.35	11.215-94.4	38.34	7.405-38.635	0.252811

Supplementary Table S2. Median serum levels of 53 cytokines, with corresponding lower limit of detection (LLOD), in patients with hepatitis B virus (HBV)-associated cirrhosis, hepatitis C virus (HCV)-associated cirrhosis, alcoholic liver disease (ALD)-associated cirrhosis, non-alcoholic fatty liver disease (NAFLD)-associated cirrhosis and healthy controls. P-values <.05 represent statistically significant cytokine differences between groups (based on Kruskal-Wallis test with Bonferroni assessment).
