# Supplementary material for: Circulating Cytokines Reflect the Etiology-Specific Immune Environment in Cirrhosis and HCC
Source: Cancers (Basel). 2022 Oct 7;14(19):4900. doi: 10.3390/cancers14194900 (PMC9563264; doi:10.3390/cancers14194900)
Supplement: Supplementary file 1 [file Supplementary Figure S3.pdf › Supplementary Figure S3.pdf]

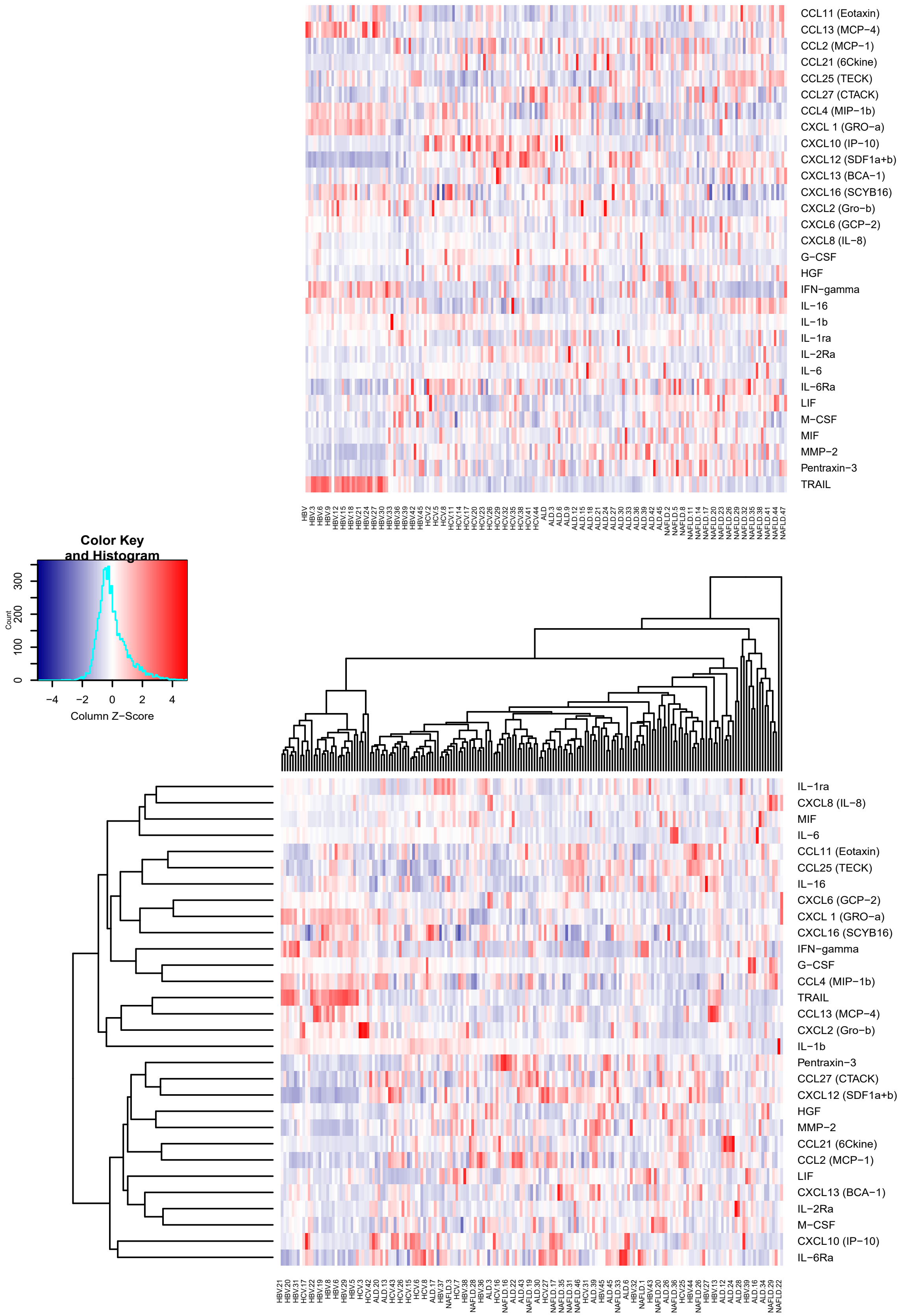

Supplementary Figure S3. Heatmaps depicting z-scores of selected serum cytokines across patients with HBV-, HCV-, ALD- and NAFLD-cirrhosis. Scaling was conducted per cytokine (row) using the mean pathway score. Positive and negative z-scores in (A) and (B) heatmaps are colored in gradients of red and blue, respectively. Heatmaps are depicted wih (A) and without (B) two-way clustering. Abbreviations: Hepatitis B virus (HBV), hepatitis C virus (HCV), Alcoholic liver disease (ALD), non-alcoholic fatty liver disease (NAFLD)
